# Clinical and pathological findings in neurolymphomatosis: Preliminary association with gene expression profiles in sural nerves

**DOI:** 10.3389/fonc.2022.974751

**Published:** 2022-09-26

**Authors:** Federica Cerri, Francesco Gentile, Ferdinando Clarelli, Silvia Santoro, Yuri Matteo Falzone, Giorgia Dina, Alessandro Romano, Teuta Domi, Laura Pozzi, Raffaella Fazio, Paola Podini, Melissa Sorosina, Paola Carrera, Federica Esposito, Nilo Riva, Chiara Briani, Tiziana Cavallaro, Massimo Filippi, Angelo Quattrini

**Affiliations:** ^1^ Experimental Neuropathology Unit, Institute of Experimental Neurology, Division of Neuroscience, IRCCS Ospedale San Raffaele Scientific Institute, Milan, Italy; ^2^ Department of Neurology, IRCCS Ospedale San Raffaele Scientific Institute, Milan, Italy; ^3^ Department of Neurology IRCCS Istituto Auxologico Italiano, Milan, Italy; ^4^ Laboratory of Human Genetics of Neurological Disorders, Institute of Experimental Neurology, Division of Neuroscience, IRCCS Ospedale San Raffaele Scientific Institute, Milan, Italy; ^5^ Division of Genetics and Cell Biology and Laboratory of Clinical Molecular Biology and Cytogenetics, Unit of Genomics for Human Disease Diagnosis, IRCCS Ospedale San Raffaele Scientific Institute, Milan, Italy; ^6^ Department of Neuroscience , University of Padova, Padova, Italy; ^7^ Department of Neurology, Azienda Ospedaliera Universitaria Integrata, University Hospital G.B. Rossi, Verona, Italy

**Keywords:** lymphoproliferative disorders, non-hodgkin lymphoma, ribosomal proteins, neuropathy, nerve biopsy

## Abstract

Although inflammation appears to play a role in neurolymphomatosis (NL), the mechanisms leading to degeneration in the peripheral nervous system are poorly understood. The purpose of this exploratory study was to identify molecular pathways underlying NL pathogenesis, combining clinical and neuropathological investigation with gene expression (GE) studies. We characterized the clinical and pathological features of eight patients with NL. We further analysed GE changes in sural nerve biopsies obtained from a subgroup of NL patients (n=3) and thirteen patients with inflammatory neuropathies as neuropathic controls. Based on the neuropathic symptoms and signs, NL patients were classified into three forms of neuropathy: chronic symmetrical sensorimotor polyneuropathy (SMPN, n=3), multiple mononeuropathy (MN, n=4) and acute motor-sensory axonal neuropathy (AMSAN, n=1). Predominantly diffuse malignant cells infiltration of epineurium was present in chronic SMPN, whereas endoneurial perivascular cells invasion was observed in MN. In contrast, diffuse endoneurium malignant cells localization occurred in AMSAN. We identified alterations in the expression of 1266 genes, with 115 up-regulated and 1151 down-regulated genes, which were mainly associated with ribosomal proteins (RP) and olfactory receptors (OR) signaling pathways, respectively. Among the top up-regulated genes were actin alpha 1 skeletal muscle (ACTA1) and desmin (DES). Similarly, in NL nerves ACTA1, DES and several RPs were highly expressed, associated with endothelial cells and pericytes abnormalities. Peripheral nerve involvement may be due to conversion towards a more aggressive phenotype, potentially explaining the poor prognosis. The candidate genes reported in this study may be a source of clinical biomarkers for NL.

## Introduction

Neurolymphomatosis (NL) is characterized by a direct invasion of the peripheral nervous system (PNS) by lymphoma, usually B-cell non-Hodgkin lymphoma (NHL) (1–5). To date, only few treatments can prolong survival of NL patients, and a diagnostic biomarker is still lacking. NL has been shown to manifest as a variety of clinical forms, including symmetrical polyneuropathy, painful polyradiculopathy, cranial neuropathies, mononeuropathies, painful or painless multiple mononeuropathies (6, 7). It is, however, uncertain which correlations exist between the histopathologic features and clinical manifestations of the NL-associated neuropathy.

Activated lymphocytes can cross the blood-nerve barrier, allowing a direct attack against one of PNS elements when an immune tolerance breakdown occurs (8). The PNS has blood-nerve-barrier composed of tight capillary endothelial junctions that are ensheathed by pericytes embedded in their basement membrane (9), which accounts for relative resistance to haematogenous spread of tumour cells. Thus, the underlying mechanisms by which malignant B-cells infiltrate the PNS and cause nerve damage are still unknown. This process needs multiple factors, including cell adhesion molecules, chemokine receptors, cytokine secretion and altered expression of extracellular matrix (ECM) components (10). Within nerve, the ECM influences axo-glia interactions, Schwann cell differentiation and the environment for axonal regeneration (11, 12). Ultimately, these mechanisms all converge on axonal preservation, regeneration and repair, which dictate the outcome of clinical symptoms (12). Several ECM components have been implicated in tumour growth, progression, and metastasis both in solid and haematological malignancies (10). Therefore, understanding the mechanisms that mediated malignant B-cell trafficking into the PNS and the interactions with the non-malignant cells and stromal elements, could provide relevant information on the pathogenesis of NL.

Taking advantage of human sural nerve biopsies, we sought to investigate the correlations between the clinical manifestations of the NL-associated neuropathy and the localization of malignant cells in the nerve. Finally, with a transcriptomic assay we investigated genes and the molecular pathways that may underlie NL pathogenesis. The characterization of these pathways might help identify new biomarkers, thus proving insight for the development of new therapeutic strategies.

## Methods

### Patients and sural nerve biopsy

We retrospectively reviewed the clinical and pathological features of 8 patients in whom the diagnosis of NL was pathological confirmed by sural nerve biopsy, showing malignant lymphocytes infiltrate, which was monoclonal on immunohistochemical (IHC) cell typing (1, 6). Clinical data were retrieved from hospital records. The presence of neuropathy was diagnosed on the basis of clinical symptoms and signs (distal sensory disturbance and weakness in the lower and, less extensively, in the upper limbs, with reduced or absent deep tendon reflexes) and electrophysiological studies (slowing of sensory and motor nerve conduction velocities with reduction of compound muscle and sensory action potentials). Patients underwent routine laboratory tests, including screening for immune-mediated and inflammatory disorders; liver, thyroid and kidney function; glucose intolerance and cerebrospinal fluid examination (CSF) with immunophenotyping applied if feasible (**Table 1**).

**Table 1:** Clinical characteristic of patients with neurolymphomatosis.

Case	Age/sex	Onset, neuropathy	Distal weakness	Proximal weakness	Pain	Sensory loss	Degree	History of haematological disorders	CSF: Protein mg/dL – Cell no/mm^3^
1	78/M	S, SMPN	U, L	L	L	L	Severe	CLL	80 - 6 (no malignant)
2	58/F	A, MN	U, L	–	L	L	Moderate	CLL	ND
3	70/M	A, MN	L	–	L	L	Moderate	CLL	NA
4	66/F	A, MN	L	L	L	L	Severe	B-NHL	NA
5	58/M	A, MN	U, L	L	L	U/L	Moderate	IgMk/CA	40 - 2 (no malignant)
6	55/M	S, SMPN	U, L	–	U, L	U, L	Severe	IgMk/Cry	NA
7	58/M	S, SMPN	L	–	L	L	Moderate	IgMk (MGUS)	NA
8	82/F	A, AMSAN	U, L	U, L	L	U, L	Severe	IgMk (MGUS)	65 - 3 (no malignant)

A, acute; S, subacute; SMPN, sensorimotor polyneuropathy; MN, multiple mononeuropathy; AMSAN, acute motor-sensory axonal neuropathy; U, upper limbs; L, lower limbs; CLL, chronic lymphocytic leukemia; B-NHL, B-cell non-Hodgkin’s lymphoma; CA, cold agglutinin; Cry, cryoglobulins; IgMk, IgM kappa; MGUS, monoclonal gammopathy of undetermined significance; CSF, cerebrospinal fluid; NA, not available; ND, not diagnostic (blood CSF contamination).

Sural nerve biopsies had been obtained for diagnostic purposes, after informed consent, and stored in our Institute of Experimental Neurology (INSPE) tissue bank. All experimental protocols were approved by San Raffaele Scientific Institute Ethical Committee (Milan, Italy), and the methods were carried out according to the approved guidelines. Sural nerve biopsies were taken and processed as previously described (13). The sural nerve was prepared using standard methods, including paraffin sections stained with haematoxylin-eosin (H&E), semithin plastic sections stained with toluidine blue and ultrathin sections for electron microscopy. IHC analysis was performed on paraffin nerve sections using the following antibodies: leukocyte common antigen (LCA), T-cells (CD3), B-cells (CD20, CD5), macrophages (CD68) markers. Antibodies anti-kappa and anti-lambda chains were used to detect B-cell monoclonality.

### RNA extraction and gene expression analysis

GE analysis was performed on RNA extracted from sural nerve diagnostic biopsies, chosen according to frozen tissue availability, obtained from 3 NL, and 13 patients with inflammatory neuropathies used as neuropathic controls, including vasculitic neuropathy (VN, n=9) and chronic inflammatory demyelinating polyradiculoneuropathy (CIDP, n=4) to discover transcriptomic changes that could account for the differences in their inflammatory pathogenesis. According to clinicalpathological and electrophysiological criteria, patients were assigned to different groups. Group 1: NL (3 cases); Group 2: CIDP (4 cases); Group 3: VN (9 cases). Total RNA was isolated from sural nerve samples using the RNeasy kit (Qiagen, Netherland). RNA was quantified using the Nanodrop-2000 spectrophotometer (Celbio, Milan, Italy). Agilent 2100 Bioanalyzer (Agilent Technologies, Palo Alto, CA) was used to assess RNA integrity. GE study was performed using the Illumina HumanHT-12 v4 BeadChips (Illumina Inc., San Diego, California, USA), targeting more than 47.000 transcripts selected primarily from the NCBI RefSeq database (Release 38). We adopted the Illumina Whole-Genome GE DASL HT Assay, which is optimized for degraded RNA (14). An amount of 250 ng of total RNA was retrotranscribed to cDNA using biotinylated oligo (dT) and random nonamer primers. The biotinylated cDNA was then annealed to the DASL Assay Pool probe groups, containing specific oligonucleotides of 50 bases designed to interrogate each target sequence in the transcripts. Then, universal PCR amplification and Cy3 staining steps were performed. Finally, the resulting labelled PCR products were hybridized to the BeadChips, which were imaged using the Illumina BeadArray Reader. The software Illumina GenomeStudio version 2011.1 was used to generate fluorescent hybridization signals.

### Pre-processing and differential expression

We performed quality controls assessing the presence of outliers by means of principal component analysis (PCA) and signal intensities distribution analysis with Bioconductor package Array Quality Metrics (15). Normalization of signals was performed with quantile procedure as available in lumi Bioconductor package (16). We retained genes deemed expressed, i.e. mapped by probes that were called ‘Present’ by GenomeStudio algorithm (detection call p-value < 0.05) on at least 50% of samples in at least one of the three groups (17). We performed PCA on the normalized filtered set of 20.418 probes. The samples were projected on the first 3 PCs, which overall explain more than 50% of the variance of expression.

Differential expression analysis was performed using moderated t-statistics as implemented in limma package (18) to detect differentially expressed genes (DEGs). Correction for multiple testing was done by controlling the False Discovery rate (FDR) with Benjamini-Hochberg procedure (19). Threshold values to declare DEGs were set to FDR < 0.05 and fold-change (FC) > 2 or FC <0.5.

### Pathway analysis

Over-representation analysis with hypergeometric test was performed interrogating Kyoto Encyclopedia of Genes and Genomes (KEGG) database through WebGestalt tool (release 17/01/2019: http://www.webgestalt.org) (20), ENCODE/ChEA and Reactome 2016 databases through EnrichR (http://amp.pharm.mssm.edu/Enrichr/enrich) (21, 22) tool pathway and functional enrichment analysis separately for up- and down-regulated DEGs, applying Benjamini-Hochberg (19) multiple testing adjustment. Input genes were provided as “gene symbol” and the filtered set of 20.418 genes as customized background set for the analysis. For WebGestalt analysis, we considered for the analysis only pathways with at least 10 and at most 400 annotated genes.

### Array data validation by Real-Time PCR and immunohistochemistry

In order to validate the microarray results, expression levels of selected DEGs was confirmed by quantitative RT-PCR performed on cDNA samples (selected from the pool of patients, three from each group: NL, CIDP and VN). *ACTA1* and *DES* were selected for validation based on having a large change in expression level (>3 fold). RT-PCR was performed using Sso Fast EvaGreen Supermix (Bio-Rad Laboratories, California, USA) as previously described (23). Briefly, three technical replicates were prepared for each sample. PCR were run on the C1000 Thermal Cycler with CFX96 Real-Time System (Bio-Rad Laboratories, California, USA). Results were analysed by CFX Manager software (Bio-Rad Laboratories, California, USA) and relative expressions were calculated with DDCt method. For each gene, the Pearson correlation coefficient was calculated between the log2-transformed expression values as measured by microarray and the negative of the Ct obtained by RT-qPCR analysis.

To verify the protein levels by IHC analysis, *ACTA1*, *DES*, *RPL26, RPS27* and *RPS29* were selected based on functions of encoded proteins and the extent of expression between the groups. All sural nerve samples were prescreened for frozen and paraffin block availability and for tissue quality and those which passed this first step were included for IHC analysis. The IHC analysis was performed as described (23) on sural nerve paraffin or cryosections from patients with NL (6 cases) and controls (2 CIDP, 4 VN). All antibodies (Abs) were tested to obtain the ideal staining conditions. The best staining conditions were: polyclonal Ab to ACTA1 (Santa Cruz, dilution 1:250), polyclonal Ab to Desmin (Novocastra, dilution 1:100), polyclonal Ab to RPL26 (Abcam, dilution 1:250) on paraffin embedded sural nerves by IHC; instead polyclonal Abs to RPS27 (ABclonal, dilution 1:100) and polyclonal anti RPS29 (Abcam, dilution 1:100) worked well on nerve cryosections using immunofluorescence staining. Double staining was performed with Ab recognizing neurofilaments (NF) to mark axons.

Double immunofluorescence on sural nerves was performed using Abs RPS27, RPS29 and NF. Sections were permeabilized for 2 min with acetone. Sections were incubated at 4°C overnight with primary Ab, washed twice with PBS for 10 min, and incubated for 30 min with FITC-conjugated or TRITC-conjugated secondary Abs, diluted 1:150 in PBS. Controls included omission of primary Abs on parallel sections.

For nerve analysis, digitalized images of nerve cross sections were obtained from sural nerve with a digital camera (Leica DFC300F) using a 40X objective of microscope (Olympus BX51). All slides were interpreted by experienced neuropathologists; at the time of examination, the pathologists were blinded to all clinical and pathological data. Interpretation of IHC staining was made independently.

## Results

### Clinical and neuropathological correlates in neurolymphomatosis

Eight patients were diagnosed with NL. The main clinical features of the 8 patients at the time of hospital admission are summarized in **Table 1**. The age at the time of nerve biopsy ranged from 55 to 82 years. Haematological disorders were present before the onset of NL in 7/8 patients, including chronic lymphocytic leukaemia (CLL, cases 1 and 3), B-cell NHL (case 4), cold agglutinin disease (CAD, case 5), chronic HCV-related hepatitis with mixed cryoglobulinemia (case 6) and IgM kappa monoclonal gammopathy of undetermined significance (MGUS) (cases 7 and 8). In patient 2, NL was the presenting symptom of an underlying CLL. Cases 2 and 5 have previously been reported (24, 25).

Clinical examination disclosed three different patterns of neuropathy: chronic symmetric sensorimotor polyneuropathy (SMPN: cases 1, 6, 7), multiple mononeuropathy (MN: cases 2, 3, 4, 5), and acute motor-sensory axonal neuropathy (AMSAN: case 8) (**Table 1**).

Light microscopic examination of transverse sections of paraffin-embedded sural nerve showed inflammatory cell infiltrates in all patients. As initial screening, LCA was an excellent cell marker for paraffin sections to reveal inflammatory infiltrates. Axonal degeneration was the predominant pathological process in all our cases.

**Figure 1:**
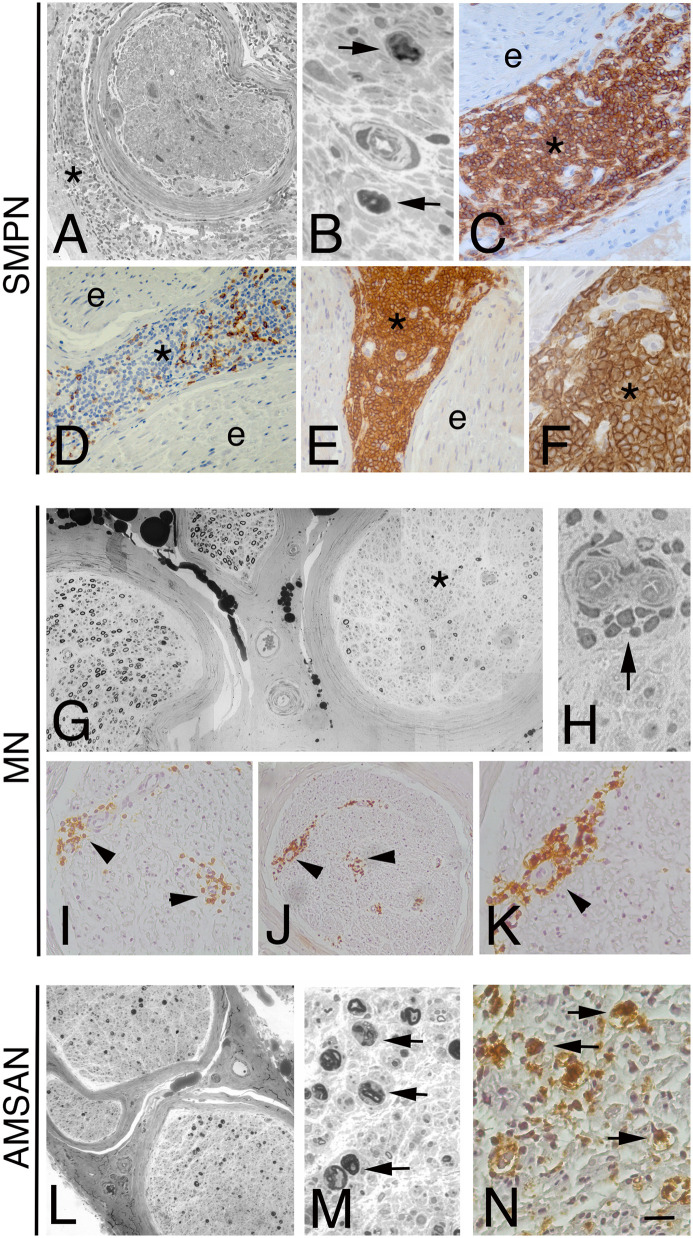
Representative neuropathological cases of NL. Transverse semi-thin sections of sural nerve from a sensorimotor peripheral neuropathy (SMPN) case (**A**, **B**): severe loss of myelinated fibres is evident in a fascicle (**A**), surrounded by lymphomatous cells in epineurium (**A**, asterisk); high magnification shows myelin ovoids, indicating axonal degeneration (**B**, arrows). LCA immunohistochemistry shows massive infiltration by large lymphoid cells in epineurium (**C**, asterisk; e marks endoneurium); anti-CD3 antibody staining shows few positive cells in epineurium (**D**, asterisk; e marks endoneurium). The majority of LCA positive cells display immunoreactivity for the CD20 B-cells marker in the epineurium (asterisk, **E**, **F**; e marks endoneurium). **(G-K)**: epoxy and paraffin sections of a sural nerve from a patient with multiple mononeuropathy (MN). Semi-thin cross-section of nerve specimen showing a non-uniform fascicular reduction of myelinated fibres, one fascicle is more severely affected (**G**, asterisk) than others. Note atypical perivascular mononuclear cells infiltrate in the endoneurium (**H**, arrow). Immunohistochemistry on paraffin section showed LCA-cells perivascular infiltrate in endoneurium (**I**, arrowheads) that immunostain for CD20 B-cell marker (**J**, **K**, arrowhead). Acute motor-sensory axonal neuropathy (AMSAN, **L**-**N**): semi-thin sections of sural nerve show reduction of myelinated nerve fibres **(L)**, without axonal regeneration. Axonal degeneration characterized by numerous myelin ovoids (**M**, arrows). Immunohistochemistry shows massive cell infiltrate in the endoneurium by malignant B-cells (CD 20+) (**N**, arrows). Scale bar represent 200µm (**A**, **D**, **E**, **G**, **I**, **J**, **L**); 50µm **(K)**; 20µm **(B, C, F, H, M, N)**.

The first subgroup consists of 3 patients with SMPN. The onset of neuropathy was sub-acute with a chronic progressive course. All patients had severe weakness, especially in the distal muscle of the legs and less frequently in the arms and thighs. The tendon reflexes were absent or reduced in the four limbs. The patients complained of paraesthesia and pain distally in the legs, and all had sensory loss in the feet. Cranial nerve involvement was not present. Electrophysiological studies showed a symmetrical sensorimotor axonal neuropathy in all cases. Patients were treated with various chemotherapy regimens. Progression of the disease led to death in 2 cases (patients 1 and 6), whereas patient 7 had neurological stabilization. Morphological examination showed a severe reduction of myelinated fibres, uniformly distributed within and between the fascicles, and signs of axonal degeneration (**Figure 1A, B**). H&E and LCA–stained paraffin sections revealed a diffuse lymphoid cell infiltrate prevalently in the epineurium in all cases (**Figure 1C**). Scarce cells infiltrate was present in the endoneurium. Paraffin immunoperoxidase staining of the LCA-positive inflammatory infiltrates showed few CD3-positive T-cells (**Figure 1D**); almost all cells were large CD20-positive B-cells (**Figure 1E, F**). In all patients, kappa light-chain-restricted of the CD20-positive B-cells was documented.

A total of 4 patients complained of MN at the onset. The initial neuropathic symptom was the acute onset of asymmetrical paraesthesia or painful dysesthesia distally in the limbs. Subsequently, patients 2, 3 and 5 developed a confluence of motor and sensory symptoms, causing an asymmetric polyneuropathy. Patient 4 presented with excruciating pain distally in right leg with severe weakness of anterior tibial muscle. In all 4 patients, electrodiagnostic studies revealed asymmetric axonal sensorimotor MN. Patients with CLL (2 and 3) and patient 4 were treated with rituximab leading to clinical improvement in cases 2 and 4, no improvement in case 3 with subsequent death. Patient 5 was treated with chemotherapy and plasmapheresis but, after an initial improvement, he died 3 months later. In sural nerve biopsies from MN cases, the reduction of fibres was focal/multifocal among and within fascicles, associated with axonal degeneration (**Figure 1G**). Perivascular inflammatory/lymphomatous infiltrate of CD20 positive B-cells in endo and sub-perineurium was observed (**Figure 1H-K**), suggesting an ischemic-mediated damage causing an axonal neuropathy with selective nerve fascicular involvement.

One patient (case 8) presented with a history of acute (7 days) progressive lower limbs weakness and back pain, preceded by a flu-like illness. Laboratory investigations showed an IgMk M-protein. CSF examination was normal. Intravenous immunoglobulin (IVIg) therapy was administered with no benefit, and the weakness progressed leading within few days to tetraparesis associated with widespread reduction of joint position sense. Neurophysiological examination was consistent with a diagnosis of AMSAN (26). Despite an additional course of IVIg therapy, the patient developed tetraplegia, sensory loss to all modalities with distal to proximal gradient. Repeated CSF was normal. A bone marrow aspirate showed an increase of small to medium size B lymphocytes (CD 19+, CD 5-, CD 23-, CD 10-; BCR-ABL -), with clonal k light chain restriction. Sural nerve biopsy showed severe reduction of the myelinated nerve fibres and axonal degeneration (**Figure 1L, M**). In the endoneurium, many medium-size lymphocytes were present. Immunophenotyping showed few scattered T (CD3 positive) cells but almost all lymphocytes were CD20+ and CD5+ B-cells (**Figure 1N**) with k light chain restriction. Combined rituximab and chemotherapy was started. Patient died of pulmonary infection 10 months after the AMSAN onset.

### Gene expression analysis

GE analysis was performed on sural nerves from NL patients to identify alterations in gene transcription associated with the disease, comparing NL samples with CIDP plus VN subjects. Indeed, clinical presentation of inflammatory neuropathies, including NL, can be similar despite a different etiology. The malignant B-lymphocytes of NL differ from the inflammatory infiltrates observed in CIDP and VN. In CIDP, the inflammatory cells include CD8+ T-cells and macrophages in endo- and epineurium. In VN, macrophages, CD4+ and CD8+ T-cells are a major component of the perivascular inflammatory infiltrate. The characteristics of patients included in GE analysis are described in **Table 2**.

**Table 2:** Characteristics of patients included in gene expression analysis.

Patients	Age	Sex	Neuropathy	Pathology	CD20	CD3	CD68
NL-1	55	M	SMNP	Severe/ax/chr	+++	0/+	0/+
NL-2	58	M	MN	Moderate/ax/ac	++	0	+
NL-3	82	F	AMSAN	Severe/ax/ac	+++	+	+
CIDP-1	51	F	CIDP	Moderate/dem/chr	0	+	+
CIDP-3	60	M	CIDP	Moderate/dem/chr	0	+	+
CIDP-4	72	M	CIDP	Severe/dem/chr	0	0/+	+
CIDP-5	65	M	CIDP	Moderate/dem/chr	0	+	+
VASC-1	65	M	VASC	Severe/ax/chr	0/+	+++	0/+
VASC-2	60	M	VASC	Severe/ax/ac	0	++	+
VASC-3	72	F	VASC	Severe/ax/ac	0	++	+
VASC-4	65	M	VASC	Severe/ax/chr	0	++	0/+
VASC-6	78	F	VASC	Severe/ax/ac	0	+++	+
VASC-7	61	F	VASC	Severe/ax/ac	0	++	+
VASC-8	58	M	VASC	Severe/ax/ac	0/+	+++	++
VASC-9	70	F	VASC	Severe/ax/ac	0/+	+++	++
VASC-10	78	M	VASC	Moderate/ax/chr	0	++	0/+

Pathological characteristic of patients with neurolymphomatosis (NL), chronic inflammatory demyelinating polyradiculoneuropathy (CIDP) and vasculitic neuropathy (VN) used for gene expression analysis. SMPN, sensorimotor polyneuropathy; MN, multiple mononeuropathy; AMSAN, acute motor-sensory axonal neuropathy; ax, axonal; Ac, acute; Chr, chronic; Dem, demyelinating; CD20, B cell marker; CD3, T cell marker; CD68, macrophage marker. Amount of inflammatory infiltrate is graded from +++ (great number of positive cells) to 0 (absence of immunoreactivity).

**Figure 2:**
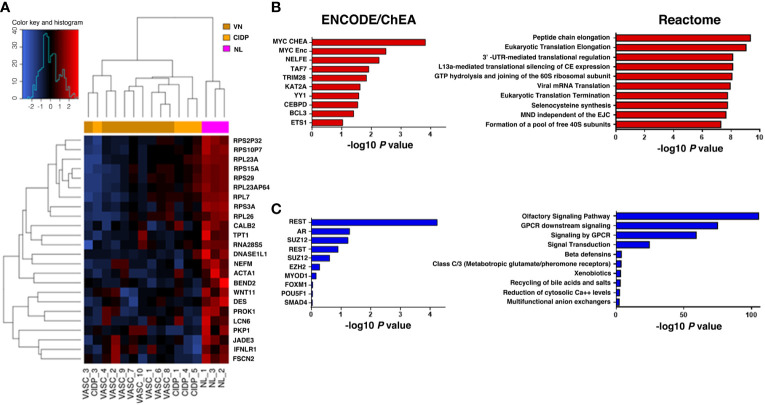
Heatmap representing hierarchical clustering of the 16 samples (columns) and gene (rows) detected as highly differentially expressed. The expression level of each gene has been standardized by subtracting the gene’s mean expression level and dividing by the standard deviation across all samples. The scaled expression value is plotted in red-blue scale colour, with red indicating higher expression and blue lower expression in NL patients **(A)**. Functional enrichment analysis on upregulated genes (**B**, red) identified *MYC* as the top ranked upstream transcription factor in our up-DEGs (ENCODE/ChEA consensus database), while enriched downstream pathways were those related to ribosomal biogenesis and mRNA translation (Reactome database). Downregulated genes (**C**, blue) were mainly associated with olfactory and GPCR signalling (Reactome database), highlighting *REST* as the most relevant transcription factor enriched in our analysis (ENCODE/ChEA consensus database).

Differential expression was assessed on the 20.418 probes that passed the filter criterion. A PCA score plot for the first 3 PCs and a bar plot with percentage of explained variance is reported in **Supplementary Figure 1** which shows a separation between NL and control samples along PC1. Limma method identified a total of 1266 DEGs: 115 were up-regulated and 1151 down-regulated in NL patients (**Figure 2A**).

A list of the most up- and down-regulated DEGs in NL patients is reported in **Table 3**. Actin, alpha 1, skeletal muscle (*ACTA1*), expressed by myoepithelial cells, skeletal and smooth muscle (27, 28), was the top up-regulated gene (FC=7.27; FDR=0.02), followed by ribosomal protein (RP) S3A (*RPS3A*) (FC=5.08; FDR=0.006) that is involved in oncogenesis (29) and the intermediate filament protein, Desmin (*DES*) (FC=4.23, FDR=0.012) expressed by smooth muscle cells and pericytes associated with blood vessels (30).

**Table 3:** Differentially expressed genes (DEGs).

Up-regulated genes						
Gene ID	Gene symbol	Name	Fold change	p-value	FDR	
ILMN_2125869	*ACTA1*	actin, alpha 1, skeletal muscle	7,2797	0,000470639	0,019718928	
ILMN_3245578	*RPS3A*	ribosomal protein S3A	5,0775	6,07537E-06	0,005987263	
ILMN_1698995	*DES*	desmin	4,2277	8,89053E-05	0,012390422	
ILMN_1690295	*RPS29*	ribosomal protein S29	3,9563	0,002075577	0,038619548	
ILMN_3277297	*RPL23AP64*	ribosomal protein L23a pseudogene 64	3,9394	0,001460856	0,032343501	
ILMN_3226301	*RPL23A*	ribosomal protein L23a	3,8881	0,000977331	0,026345642	
ILMN_3287033	*RPS10P7*	ribosomal protein S10 pseudogene 7	3,8641	6,6102E-05	0,011852474	
ILMN_3267476	*RPS15A*	ribosomal protein S15a	3,6941	0,001624654	0,034331835	
ILMN_3213692	*RPL26*	ribosomal protein L26	3,6513	0,002744187	0,044827882	
ILMN_3208014	*RPS2P32*	ribosomal protein S2 pseudogene 32	3,6100	0,000142745	0,014280156	
ILMN_3246805	*RNA28S5*	RNA, 28S ribosomal 5	3,5872	0,002388714	0,041652457	
ILMN_1663454	*PKP1*	plakophilin 1	3,5424	0,00268466	0,044387461	
ILMN_1789614	*TPT1*	tumor protein, translationally-controlled 1	3,5292	0,00046695	0,019718928	
ILMN_2092390	*BEND2*	BEN domain containing 2	3,5284	0,000129398	0,013901056	
ILMN_3249756	*DNASE1L1*	deoxyribonuclease I-like 1	3,3866	3,18862E-05	0,00903084	
ILMN_1658531	*RPL7*	ribosomal protein L7	3,3625	0,000909295	0,025488307	
ILMN_1792800	*LCN6*	lipocalin 6	3,3435	0,002461731	0,042372083	
ILMN_1662188	*WNT11*	wingless-type MMTV integration site family, member11	3,2129	0,002557215	0,04338193	
ILMN_2405324	*IFNLR1*	interferon, lambda receptor 1	3,1911	0,00143763	0,032051291	
ILMN_1713247	*FSCN2*	fascin actin-bundling protein 2, retinal	3,1491	0,000462448	0,019718928	
ILMN_2383807	*CALB2*	calbindin 2	3,1219	0,001070951	0,027444528	
ILMN_2215989	*NEFM*	neurofilament, medium polypeptide	3,1117	0,000445904	0,01944853	
ILMN_2279144	*JADE3*	jade family PHD finger 3	3,0887	0,002923497	0,046300381	
ILMN_1716259	*PROK1*	prokineticin 1	3,0772	0,000430043	0,019278224	
**Down-regulated genes**						
**Gene ID**	**Gene symbol**	**Name**	**Fold change**	**p-value**		**FDR**
ILMN_1658386	*DAOA*	D-amino acid oxidase activator	0,0621	3,06967E-05		0,008991747
ILMN_1815093	*OR6M1*	olfactory receptor, family 6, subfamily M, member 1	0,0642	6,66619E-05		0,011852474
ILMN_1729963	*KCNH5*	potassium voltage-gated channel, subfamily H, member5	0,0648	6,88625E-05		0,011852474
ILMN_1727975	*SNTG1*	syntrophin, gamma 1	0,0676	0,000241646		0,01687964
ILMN_1685596	*PNLIP*	pancreatic lipase	0,0685	0,000358538		0,018661101
ILMN_1690200	*OR4F4*	olfactory receptor, family 4, subfamily F, member 4	0,0699	0,000260407		0,016992106
ILMN_1792400	*CSN3*	casein kappa	0,0714	0,001731427		0,035421154
ILMN_1804722	*OR2J3*	olfactory receptor, family 2, subfamily J, member 3	0,0721	9,25511E-05		0,012614712
ILMN_1713602	*OR14J1*	olfactory receptor, family 14, subfamily J, member 1	0,0721	0,000170715		0,015310874
ILMN_1755897	*UGT2B7*	UDP glucuronosyltransferase 2 family, polypeptide B7	0,0722	0,000210029		0,016124845
ILMN_3244688	*UGT2A2*	UDP glucuronosyltransferase 2 family, polypeptide A2	0,0731	0,000205047		0,016124845
ILMN_1797475	*FAM216B*	family with sequence similarity 216, member B	0,0740	5,82176E-05		0,011220007
ILMN_1694080	*ZNF804B*	zinc finger protein 804B	0,0742	1,93148E-05		0,007139271
ILMN_1805062	*OR51V1*	olfactory receptor, family 51, subfamily V, member 1	0,0765	0,000470258		0,019718928
ILMN_2171733	*OR5AR1*	olfactory receptor, family 5, subfamily AR, member 1	0,0767	2,77423E-05		0,008768177
ILMN_2162819	*UGT2B11*	UDP glucuronosyltransferase 2 family, polypeptide B11	0,0773	8,63887E-06		0,006077306
ILMN_3308813	*MIR599*	microRNA 599	0,0778	0,000390661		0,018798627
ILMN_3238767	*PCAT18*	prostate cancer associated transcript 18	0,0783	0,000116267		0,013415661
ILMN_1793403	*OR2G3*	olfactory receptor, family 2, subfamily G, member 3	0,0792	0,00016534		0,015078825
ILMN_1802701	*OR4N3P*	olfactory receptor, family 4, subfamily N,member 3 pseudogene	0,0817	7,73584E-06		0,005987263
ILMN_2342651	*OR11H12*	olfactory receptor, family 11, subfamily H, member 12	0,0823	0,000154282		0,014692468
ILMN_2296439	*PDE4B*	phosphodiesterase 4B, cAMP-specific	0,0824	1,08525E-05		0,006574198
ILMN_1680886	*OR5H15*	olfactory receptor, family 5, subfamily H, member 15	0,0835	1,02826E-05		0,006574198
ILMN_1793962	*OR8K5*	olfactory receptor, family 8, subfamily K, member 5	0,0853	0,000135647		0,014113166
ILMN_3242181	*SYT14P1*	synaptotagmin XIV pseudogene 1	0,0864	1,71447E-05		0,006841497
R: False Discovery Rate						

The most down-regulated gene was d-amino acid oxidase activator (*DAOA*) (FC=0.062, FDR =0.009) involved in the glutamate receptor activation and it has been shown to be associated with schizophrenia (31). The second most down-regulated gene was olfactory receptor, family 6, subfamily M, member 1 (*OR6M1*) (FC=0.064, FDR =0.011).

### Pathway analysis

DEGs were classified into functional groups according to enrichment analysis. KEGG annotation revealed two terms respectively enriched in up and down NL-related DEGs, ‘ribosome protein’ (*RPS3A, RPS29, RPL23A, RPS15A, RPL26, RPL7, RPS27, RPL21, RPL12, RPL6*) and ‘Olfactory transduction’ (*OR6M1, OR4F4, OR2J3, OR14J1, OR51V1, OR2G3, OR5H15, OR8K5, OR5AR1, OR2L2*) (**Table 4**; **Supplementary Figure 2**).

**Table 4:** Pathway analysis enrichment results on KEGG.

	Pathway	ID	Statistic	10 top annotated DEGs
Terms enriched with up-regulated genes	Ribosome	hsa03010	Ratio: 18.26 P value: 7.61E-12 adj P value: 2.37E-09	*RPS3A, RPS29, RPL23A, RPS15A, RPL26, RPL7, RPS27, RPL21, RPL12, RPL6*
Terms enriched with down-regulated genes	Olfactory transduction	hsa04740	Ratio: 6.415 P value: <0.0001 Adj P value: <0.0001	*OR6M1, OR4F4, OR2J3, OR14J1, OR51V1, OR2G3, OR5H15, OR8K5, OR5AR1, OR2L2*

Statistics according to WebGestalt. Ratio, ratio between the number of DEGs in the pathway and the number of DEGs; P-value, p-value for hypergeometric test; adj P-value, p-value for hypergeometric test adjusteted with Benjamini-Hochberg procedure.

Looking closer to upregulated genes, *MYC*, a master regulator of cell survival, is one of the most representative transcription factor gene according to the ENCODE/ChEA consensus database (FDR=0.045) and downstream effects, investigated through the Reactome 2016 database, affected mostly pathways related to the translational machinery, such as peptide elongation, termination, 3’ -UTR-mediated regulation, mRNA decay and ribosomal complex assembly (**Figure 2B**; **Table 5**). Interestingly, viral mRNA translation ranked as one of the top enriched pathways emerged from up-regulated genes, suggesting that infectious-related mechanisms may be involved in lymphoma.

**Table 5:** Pathway analysis on EnrichR of upregulated genes.

Index	Name	*p*-value	*q*-value	OR	Combined score
Top 10 enriched terms on ENCODE/ChEA consensus database					
1	MYC CHEA	0,0001482	0,01541	3,34	29,42
2	NELFE ENCODE	0,005354	0,1856	3,77	19,72
3	KAT2A ENCODE	0,02352	0,4077	4,9	18,38
4	TRIM28 CHEA	0,01447	0,301	3,5	14,83
5	MYC ENCODE	0,003164	0,1645	1,94	11,17
6	TAF7 ENCODE	0,01216	0,3161	2,3	10,13
7	YY1 CHEA	0,02612	0,3881	2,66	9,69
8	BCL3 ENCODE	0,03893	0,4049	2,69	8,74
9	CEBPD ENCODE	0,02839	0,369	2	7,14
10	ETS1 ENCODE	0,09047	0,5881	2,83	6,8
Top 10 enriched pathways on Reactome 2016 Human database					
1	Peptide chain elongation R-HSA-156902	4,44E-10	6,80E-07	21,01	597,52
2	Eukaryotic Translation Elongation R-HSA-156842	9,07E-10	6,94E-07	19,83	549,8
3	Viral mRNA Translation R-HSA-192823	1,16E-08	2,96E-06	19,26	484,89
4	Selenocysteine synthesis R-HSA-2408557	1,72E-08	3,76E-06	18,59	460,85
5	Eukaryotic Translation Termination R-HSA-72764	1,72E-08	3,29E-06	18,59	460,85
6	Nonsense Mediated Decay (NMD) independent of the Exon Junction Complex (EJC) R-HSA-975956	2,22E-08	3,09E-06	18,18	445,88
7	L13a-mediated translational silencing of Ceruloplasmin expression R-HSA-156827	7,63E-09	3,89E-06	16,65	426,18
8	3’ -UTR-mediated translational regulation R-HSA-157279	7,63E-09	2,92E-06	16,65	426,18
9	GTP hydrolysis and joining of the 60S ribosomal subunit R-HSA-72706	8,54E-09	2,61E-06	16,49	420,33
10	Formation of a pool of free 40S subunits R-HSA-72689	5,15E-08	6,06E-06	16,85	399,19

(Statistics according to EnrichR).

Detailed analysis of downregulated genes revealed that *REST*, a transcription repressor of neural-specific genes, outreached significance as the most representative upstream factor (FDR=0.0045). Reactome analysis predicted a significant loss of function in pathways related to olfactory signalling and GPCR signalling (**Figure 2C**; **Table 6**).

**Table 6:** Pathway analysis on EnrichR of downregulated genes.

Index	Name	*p*-value	*q*-value	OR	Combined score
Top 10 enriched terms on ENCODE/ChEA consensus database					
1	REST ENCODE	0,0000579	0,006022	1,9	18,8
2	SUZ12 ENCODE	0,05875	1000,0	1,8	5,0
3	AR CHEA	0,05141	1000,0	1,2	3,6
4	REST CHEA	0,1249	1000,0	1,1	2,4
5	SUZ12 CHEA	0,2426	1000,0	1,1	1,5
6	EZH2 CHEA	0,5243	1000,0	1,0	0,7
7	MYOD1 ENCODE	0,6843	1000,0	0,9	0,3
8	POU5F1 CHEA	0,9057	1000,0	0,7	0,1
9	SMAD4 CHEA	0,9123	1000,0	0,8	0,1
10	FOXM1 ENCODE	0,8949	1000,0	0,6	0,1
Top 10 enriched pathways on Reactome 2016 Human database					
1	Olfactory Signaling Pathway R-HSA-381753	5,94E-106	9,09E-103	7,63	1901,15
2	GPCR downstream signaling R-HSA-388396	1,40E-75	1,07E-72	4,13	739,56
3	Signaling by GPCR R-HSA-372790	8,37E-60	4,27E-57	3,29	470,72
4	Signal Transduction R-HSA-162582	3,60E-25	1,38E-22	1,95	123,34
5	Recycling of bile acids and salts R-HSA-159418	0,000885	0,1505	6,14	43,19
6	Multifunctional anion exchangers R-HSA-427601	0,004726	0,5562	7,9	42,3
7	Xenobiotics R-HSA-211981	0,000505	0,09653	5,53	41,98
8	Beta defensins R-HSA-1461957	0,000154	0,0471	4,37	38,33
9	Reduction of cytosolic Ca++ levels R-HSA-418359	0,003002	0,4175	6,14	35,69
10	Class C/3 (Metabotropic glutamate/pheromone receptors) R-HSA-420499	0,000234	0,05118	4,15	34,67

(Statistics according to EnrichR).

### RT-PCR and immunohistochemistry

**Figure 3:**
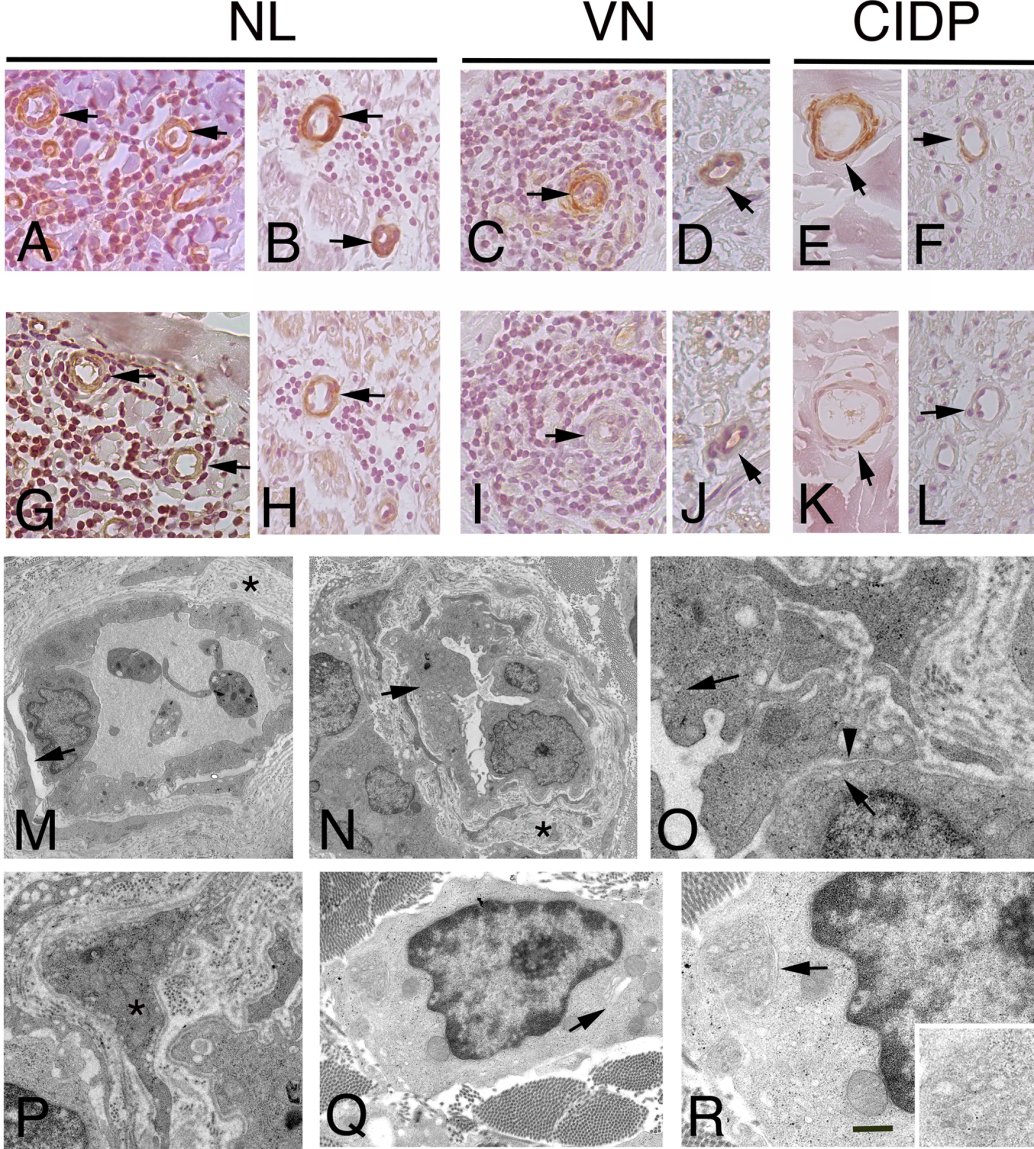
Immunohistochemistry localization of ACTA1 and desmin in NL and control nerves. ultrastructural analysis of endoneurial blood vessels in NL. Staining for ACTA1 shows increase expression in NL sural nerves. ACTA1 immunoreactivity was present in the epineurium **(A)**, arrows and endoneurium **(B)**, arrows in endothelial cells and pericytes-like cells in the proximity of malignant B-cells in patients with SMPN **(A)** and MN **(B)**. Similarly, ACTA1 shows a weak immunoreactivity in both epineurial **(C**, **E)**, arrows and endoneurial blood vessels **(D**, **F)**, arrows in control nerves. Desmin shows immunoreactivity in endothelial cells and pericytes-like cells in the epineurium and endoneurium in NL nerves from patients with SMNP **(G)**, arrows mark blood vessels in epineurium) and MN **(H)**, arrow marks an endoneurial blood vessel; in VN and CIDP sural nerves, immunoreactivity was almost negative or weak in epineurial and endoneurial blood vessels **(I**-**L)**, arrows. In NL sural nerves, electron micrographs showing abnormal endothelial cells and pericytes on blood vessels in sural nerve of patients with NL. Initial detachment of pericytes from endothelial cells **(M)**, arrow. Endoneurial capillaries showing hypertrophic endothelial cells **(N)**, arrow occluding the lumen. The basement membrane at the outer surface of the endothelial cells is increased in thickness asterisks, **(M, N)** and reduplicated; endothelial cells showing increase pinocytic vesicles **(O)**, arrows and loss of endothelial thigh junctional integrity **(O)**, arrowhead. Abnormal pericytes dropout from the endothelial cells **(P)**, asterisk. Malignant B-cells in the endoneurium between fibrotic scar **(Q)**. Note the cytoplasm with virtually no rough endoplasmic reticulum and the granularity of the cytoplasm results from the numerous polyribosomes. A small Golgi complex **(Q)**, arrow is present in one tumour cell and an abnormal residual rough endoplasmic reticulum **(R)**, arrow and high magnification). Scale bar represent 50µm **(A**-**L)**; 2μm **(M**, **N)**; 1μm **(P**, **Q)**, 500nm **(O**, **R)**.

For a subset of transcripts, quantitative RT-PCR and IHC were used to corroborate and validate microarray results. The results of quantitative RT-PCR validation, performed on three randomly selected samples for each group, confirmed a significant difference in the expression of *DES*. Although *ACTA1* was overexpressed in NL, the difference was not considered significant (**Supplementary Figure 3**). To establish the localization and the pattern of expression of ACTA1, Desmin and selected RPs in the PNS, we performed IHC analysis on cross sections of human sural nerves. We showed a correlation between mRNA and protein levels for all selected proteins, displaying elevated signal in the sural nerves from patients with NL compared with the nerve from CIDP and VN patients. ACTA1 immunoreactivity was present in both blood vessels wall in the epineurium and endoneurium of NL sural nerves (**Figure 3A, B**); weak expression was also observed in blood vessels in control nerves (**Figure 3C-F**). The expression of ACTA1 was mainly distributed in the cytoplasm of endothelial and pericytes-like cells. Similarly, both the nuclei and cytoplasm of pericytes-like cells of the epineurial and endoneurial blood vessels were positive for Desmin in NL (**Figure 3G, H**); whereas blood vessels from VN and CIDP sural nerves showed an almost negative immunoreactivity (**Figure 3I-L**).

Most vessels in NL were enveloped by cells with both ACTA1 and Desmin immunoreactivity, identifying pericytes-like cells. Thus, to determine the role of pericytes in NL, we characterized vascular structure in NL by electron microscopy, assuming that blood vessel stability might play a role in NL, depending on pericyte integrity. Electron microscopy revealed the presence of ultrastructural abnormalities (**Figure 3M-P**) in endoneurial blood vessels. Pericytes showed detachment of endothelial cells and the continuity of basement membrane was lost (**Figure 3M, N**). Hypertrophic endothelial cells were observed, showing increase endothelial transcytosis and abnormalities of thigh junctions with gaps between cells (**Figure 3O**). Pericytes changed their normal morphology, showing a migrating phenotype (**Figure 3P**). In addition, in perivascular space, malignant B-cells were found, lacking intercellular junctions and presenting many polyribosomes in the cytoplasm (**Figure 3Q, R**).

**Figure 4:**
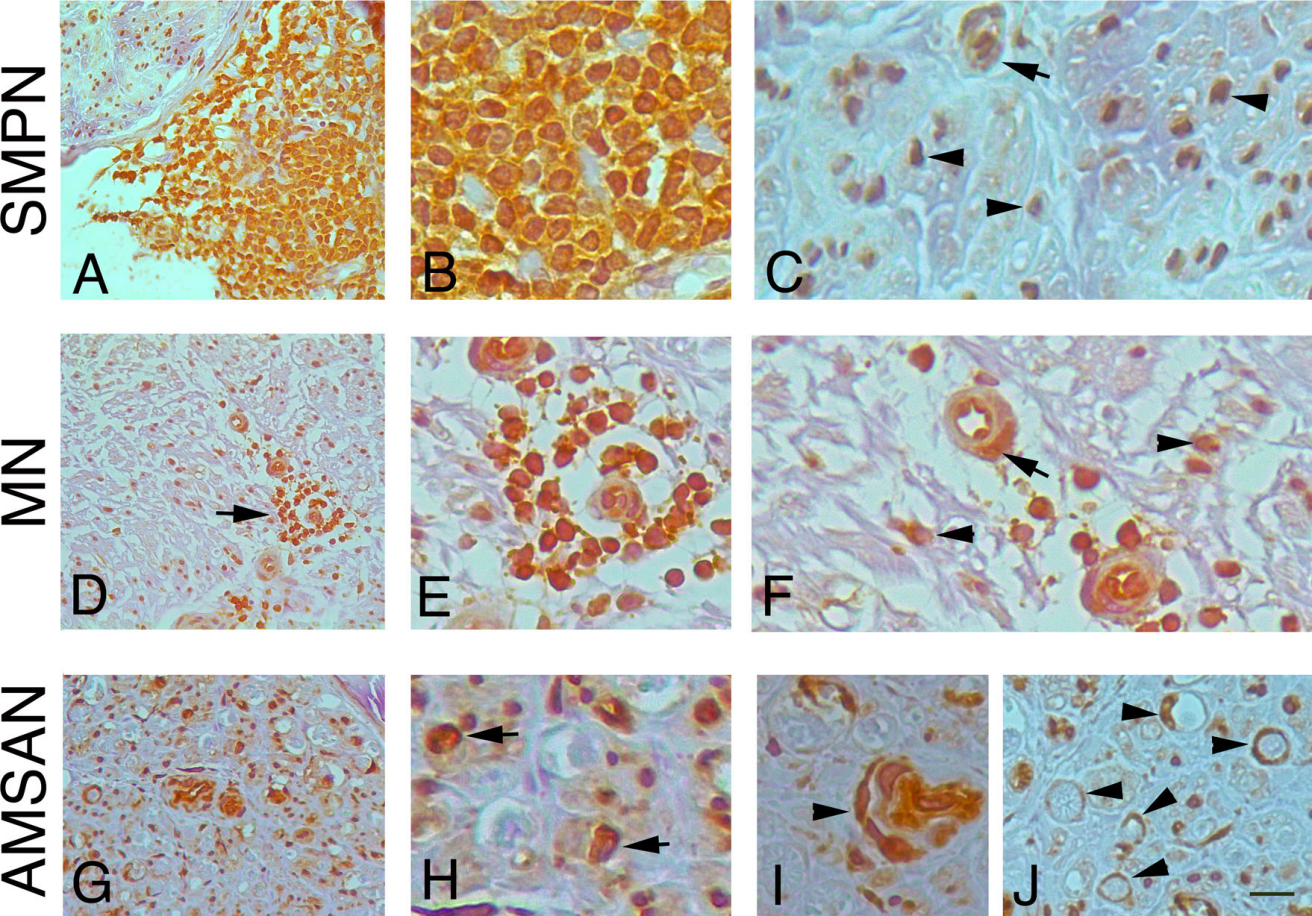
RPL26 expression in NL sural nerves. RPL26 immunoreactivity shows increase expression in NL sural nerves. RPL26 shows strong immunoreactivity of the malignant B-cells in the epineurium in SMPN **(A, B)**, endoneurial neuclei **(C)**, arrowheads and vessel **(C)**, arrow. In MN, strong immunoreactivity of perivascular malignant B-cells in the endoneurium (**D**, **E**, arrow) was present; endothelial cells, pericytes (**F**, arrow) and endoneurial cells were also positive (**F**, arrowheads). In AMSAN, strong immunoreactivity was present in endoneurium (**G**, **H**, arrows) in numerous cells, including endothelial cells and pericytes (arrowhead, i); RPL26 was associated with the rim of myelin sheets of Schwann cells (**J**, arrowheads). Scale bar represent 20µm **(A, D, G)**; 10μm **(B, C, E, F, H-J)**.

**Figure 5:**
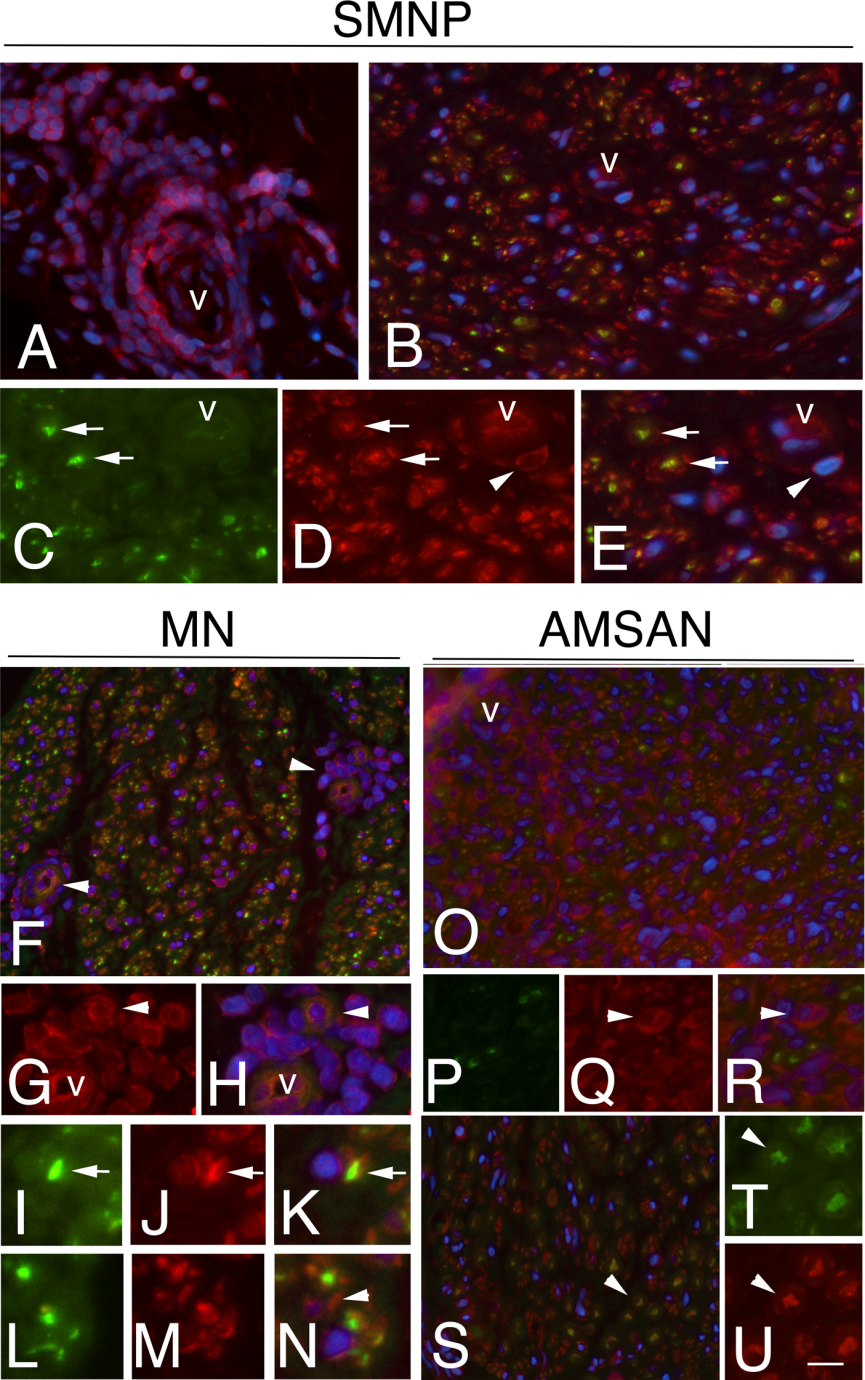
RPS27 expression in sural nerves of NL. Transverse sural nerves sections from SMPN, MN and AMSAN double-stained for RPS27 (red) and neurofilament (NF, green), to recognize axons. DAPI staining of nuclei (blue). Co-expression of NF and RPS27 by the same cell results in overlapping of the fluors, and appears yellow. SMNP: RPS27 immunoreactivity was present in epineurial malignant B-cells (**A**, v marks an epineurial vessel) and in endoneurium **(B)**; RPS27 shows expression in axons (arrows, **C**, **D**, merge in **E**) and Schwann cells (**D, E**, arrowheads). MN: RPS27 immunoreactivity of perivascular malignant B-cells in endoneurium (**F-H**, arrowhead, v marks a vessel), axons (**I-K**, arrows) and Schwann cells (**l-N**, arrowhead). RPS27 shows a diffuse increase expression in the endoneurium of nerve from a patient with AMSAN, including malignant B-cells **(O-R** arrowhead) (**O**, v marks a vessel) and axons (**S**-**U**, arrowheads). Scale bar represent 20μm **(A, B, F, O, R)**; 10μm; **(C-E, G-N, S-U)**.

**Figure 6:**
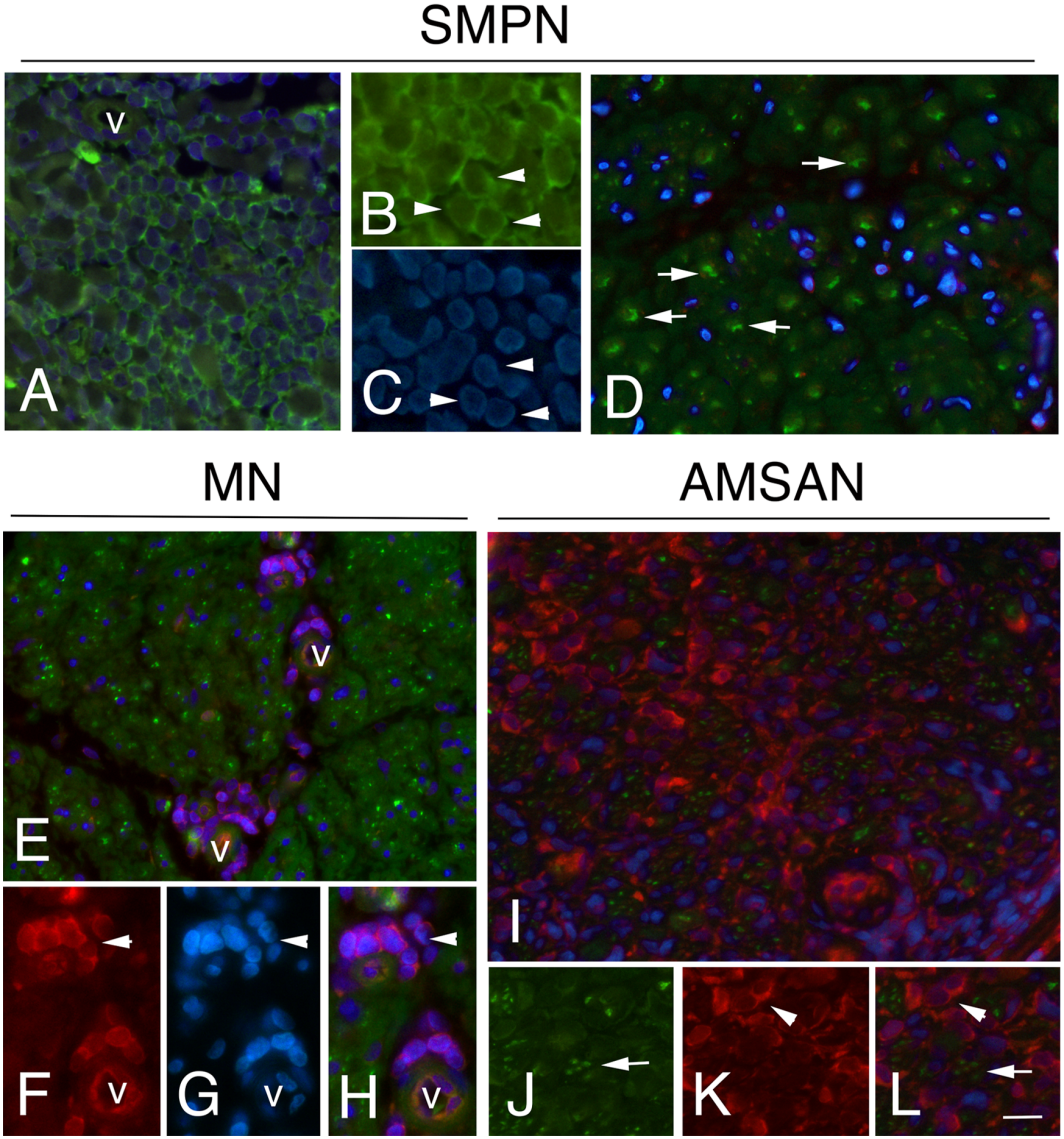
RPS29 expression in sural nerves of NL Representative micrographs of immunohistochemical staining with RPS29 marker in. NL. In all cases, RPS29 was express by malignant B-cells. **(A-C)** positive malignant B-cells in epineurium in SMPN (arrowheads, in **A** v marks a vessel); axons (green) (**D**, arrows) showing the absence of RPS29 (red) staining. **(E-H)**: RPS29 (red) perivascular positive malignant B-cells in endoneurium in MN (**F-H**, arrowhead, v marks endoneurial vessels) with negative axons (green). **(I-L)** diffuse RPS29 immunoreactivity in patients with AMSAN **(I)**: positive B-cells in endoneurium (**K, l**, arrowheads), axons were negative for RPS29 (**J**-**L**, arrows). Scale bar represent 20μm **(A, D, E, I)**; 10μm **(B, C, F-H, J-L)**.

To further examine whether the GE alterations identified by the microarray analysis also occur at the protein level, we performed IHC analysis for selected RPs, including RPL26, that has been associate with Diamond-Blackfan anaemia and tumorigenesis (32, 33); RPS27 overexpresses in several tumours and RPS29, which exhibits cell-specific expression in lymphoid cell types (34–36). **Figures 4**-**6** and **Supplementary Table 1** show the results of the IHC staining of nerve biopsies with antibodies to RPs. In SMPN, RPL26 immunoreactivity was present in nuclei and cytoplasm of malignant B-cells, localized in epineurium (**Figure 4A, B**) and cell nuclei of endoneurial cells (**Figure 4C**). In MN patients, perivascular endoneurial malignant B-cells, endoneurial cells, endothelial cells and pericytes-like cells showed high expression of RPL26 (**Figure 4D-F**). Diffuse immunoreactivity was observed in AMSAN (**Figure 4G-J**). Notably, numerous endoneurial cells, including endothelial cells, pericytes and Schwann cells showed strong RPL26 staining (**Figure 4H-J**). In VN and CIDP control nerves, RPL26 staining was weak on endothelial cells of small blood vessels and inflammatory T-cells (**Supplementary Table 1**). All malignant B-cells were positive for RPS27 (**Figure 5A-U**) and it’s expression was also in axons and Schwann cells cytoplasm, whereas there was no significant staining in CIDP and VN nerves (**Supplementary Table 1**). RPS29 was highly expressed in malignant B-cells in all NL patients (**Figure 6A-L**).

## Discussion

In this study, we described clinicopathological correlations in NL-associated neuropathy, showing that different clinical presentation may have divergent histopathological features, according to the extent of axonal damage, the distribution of the cellular infiltrates and the immunophenotype of malignant cells. We identified potential candidate markers of NL in sural nerve biopsy as well as blood-nerve-barrier abnormalities, involving pericytes and endothelial cells, which may be an important culprit driving the invasion of lymphomatous cells in the nerve.

The rarity and heterogeneous presentation of NL pose important challenges in diagnosis and management of these patients (1, 37). Given the diagnostic uncertainty, sural nerve biopsy often remains the only tool available to make a definite diagnosis, showing malignant lymphocytes infiltrate which may be located in the perineurium, epineurium, and endoneurium (2, 6). This neural involvement may result in a wide range of clinical manifestations depending upon malignant cell location in the PNS.

In our series, we observed three main clinical patterns of NL: chronic SMPN, MN and AMSAN. Diffuse and severe fibres loss was observed in chronic SMPN, whereas predominantly focal fibres loss occurred in MN. In addition, SMPN patients showed malignant cells invasion mainly in the epineurium, inducing axonal degeneration without or scarce malignant cells invasion in the endoneurium. MN was more closely associated with endoneurial malignant cells localized preferentially in the perivascular space, suggesting an ischemic process. Finally, one patient developed an AMSAN, which is a clinical and immunopathological variant of Guillian-Barré-syndrome (26), presenting rapid and severe motor and sensory dysfunction with a poor prognosis. AMSAN was described in a patient who underwent chemotherapy for Burkitt-like lymphoma (38), however sural nerve biopsy showed axonal degeneration without infiltration of malignant cells, suggesting that the damage was immune-mediated and/or due to the toxicity of vincristine. Although we cannot draw definitive conclusion from this single case, the unusual clinical picture was associated with striking findings at nerve biopsy, revealing diffuse malignant B-cells in the endoneurium, whose immunophenotype was more aggressive compared to this observed in bone marrow.

No previous study investigated GE profiles in sural nerves of NL patients, therefore, no direct comparison is possible. The findings from GE analysis provided hints on the possible mechanisms underlying local invasion of lymphoma cells. ACTA1 and DES were overexpressed in NL, prevalently in pericytes-like cells that showed well-documented ultrastructural alterations in the areas of malignant B-cell invasion. Pericytes are specialized cells located at the abluminal surface of capillary blood vessels, embedded within the basement membrane of endothelial cells (39). They regulate microvascular blood flow, are essential components of the blood-brain-barrier/blood-nerve-barrier and regulate endothelial cell proliferation during angiogenesis (39). In the tumoral context, platelet derived growth factor (PDGF)-beta signalling controls pericytes recruitment on endothelial cells with consequent high coverage of the new tumour blood vessel, promoting vascular stability and tumour growth (40, 41). It has been reported that imatinib, a tyrosine kinase inhibitor of PDGFRβ, induced apoptosis of tumour-associated PDGFRβ1 pericytes and impaired growth of lymphoma, indicating that pericytes may represent a novel target for lymphoma therapy (42). However, pericytes are critical for regulation of blood vessel integrity and permeability. Notably, ultrastructural changes of endothelial cells and pericytes has been observed in primary central nervous system lymphoma (PCNSL) (43). Low pericytes coverage with detachment from endothelial cells could compromise blood vessels structure, which becomes permeable, facilitating tumour cell invasion/extravasation and contribute to metastasis (41, 44).

Alterations in blood-nerve-barrier permeability may be favoured by pre-existing conditions such as chronic auto-immune/inflammatory diseases or haematological disorders (37), which negatively impact on the nerve microenvironment. Half of our 8 NL patients had an IgMk M-component, a known cause of peripheral neuropathy (45). CAD and cryoglobulinemia have also been associated with peripheral nerve injury through blood-nerve-barrier alterations and inflammatory mechanisms (8). These factors may favour neurotropism of B-cells and transformation into an aggressive disorder in the PNS, highlighting that nerve environment can facilitate malignant lymphoproliferation.

Pathway analysis showed a cluster of RPs among the most up-regulated genes in NL patients. Thanks to pathway analysis approach, we observed that *MYC* ranked as one of the most important transcription factor linked to RPs. *MYC* is a master regulator of cell survival in immune cells, and its overexpression is frequently observed in aggressive forms of lymphoma, such as diffuse large B cell lymphoma (DLBCL) and PCNSL (46). Furthermore, *MYC* activation underlies CLL transformation in Richter’s syndrome (47), supporting its involvement in the switch of cancerous lymphocytes towards a more aggressive phenotype.

Downstream, the upregulation of RPs implied enrichment of numerous pathways related to ribosome biogenesis and translational machinery. Dysregulation of different RPs may change the dynamics of the ribosomal complex function and the translation of wide arrays of proteins. In addition, they exert extra-ribosomal functions, participating in cell proliferation, migration, invasion, and neoplastic transformation. Indeed, RPs alterations are common in leukemic patients (48) and have been reported in several models of *MYC*-induced lymphoma (49). In DLBCL, RPs expression is associated with immunochemotherapy resistance and poor clinical outcomes (50). Besides lymphoma, raised free levels of several RPs (RPS15A, RPL23A, RPS26 and RPS29) have been associated with aggressiveness and bad prognosis also in other malignancies (51, 52). Inhibition of the p53-MDM2 axis seems pivotal for the antiapoptotic function of RPs, although the mechanisms governing this effect are poorly understood (53).

RPS29 upregulation was observed only in lymphoma cells, suggesting a targeted function in oncogenesis. Notably, RPS29 was identified to be highly associated with the expression level of B-cell translocation gene 1 (BTG1) to induce the development of DLBCL (54). Furthermore, it is a top interactor of RPS6, a key marker of mTOR pathway activation in DLBCL (55).

Overexpression of RPL26 and RPS27 was present ubiquitously in NL nerves, both in malignant and non-malignant cells. Besides lymphocytes, changes in ribosomal machinery of surrounding supportive elements, such as endoneurial and Schwann cells, may be necessary to create a tumour microenvironment able to sustain lymphoma growth. RPL26 and RPS27 have been previously reported as either prognostic factor or associated to growth regulation and tumorigenesis (32–34). Interestingly, RPS27 has been found to be highly expressed in gastric cancer tissue and cancer cells (36), correlating with the expression of integrin ß4 that has been demonstrates to promote invasion and migration in various tumours. Mutations in RPs sharply increases the incidence of malignant peripheral nerve sheath tumours in zebrafish through inhibition of p53 expression, further suggesting that RPs may tune the tumorigenic potential of peripheral nerve elements (56).

NL patients showed that the most down-regulated genes were related to ORs. A large number of ORs are expressed by Schwann cells in peripheral nerves (57). Interestingly, the OR pathway has recently emerged as a potential factor contributing to PNS nerve regeneration after nerve injury (58). Such findings may at least partially explain the poor prognosis observed in NL-related neuropathy due to progressive axonal loss without axonal regeneration. Furthermore, *REST* was the outmost significant transcription factor related to OR downregulation. Increased *REST* expression mediates injury-induced chronic pain hypersensitivity in sensory nerve fibres, by altering expression of muscarinic receptors and potassium channels (59, 60). A similar mechanism is plausible in NL, as pain is a common feature in NL patients. Alternatively, *REST* may be associated with oncogenesis, as changes in its expression have been associated with several types of cancer (61).

This study present limitations. The small number of patients does not allow coverage of the broad spectrum of clinical presentations in NL, including plexopathies and mononeuropathies, and therefore our findings cannot be generalised to the NL populations. However, the parallelisms observed between clinical-histopathological features and GE analysis point towards the existence of common and at the same time diverging mechanisms in NL, fuelling the need of more studies to confirm our results. The lack of normal control nerves, as well as the heterogeneity in sub-group samples for GE analysis may be another drawback of our study. Nonetheless, nerves from patients with inflammatory neuropathies were chosen to directly compare polyclonal and monoclonal lymphocytes’ population, filtering out genes associated with unspecific processes related to nerve injury. Furthermore, IHC validation of selected DEGs, such as ACTA1, desmin and RPs, gave results consistent with the microarray data, gaining more details on their localization and expression in nerves from NL patients and on the potential role of blood-nerve barrier in neurotropism.

## Conclusion

NL is a very heterogeneous disorder, showing several clinical presentations and different outcomes. This pilot study allows a first look into the complex of microenvironment of the sural nerve in NL that could give proliferative signals to the malignant cells, providing a source for future investigations, which may lead to the identification of potential biomarkers for NL.

## Data availability statement

The data presented in the study are deposited in the GEO repository, accession number GSE213455.

## Ethics statement

The studies involving human participants were reviewed and approved by San Raffaele Scientific Institute Ethical Committee (Milan, Italy). The patients/participants provided their written informed consent to participate in this study.

## Author contributions

FCe, NR and AQ conceived and designed the study. NR, FCl, SS, MS, CB, MF and AQ contributed on general discussion of results. FCe, FG, SS, FCl, MS and AQ had a major role in preparing the manuscript submitted for publication and generation of the figures. FCe, TC and AQ participated in neuropathological data collection and interpretation. FE, FCl, PC and AQ oversaw all the experimental procedure. GD, AR, TD, LP and PP completed the experimental protocols and had input in writing the manuscript. YF, RF, CB and MF provided clinical expertise, patient selection and clinical data collection. All authors contributed to data interpretation. FCe and FG wrote the first draft of the article. All authors contributed to the article and approved the submitted version.

## Conflict of interest

MF is Editor-in-Chief of the Journal of Neurology and Associate Editor of Neurological Sciences, received compensation for consulting services and/or speaking activities from Bayer, Biogen Idec, Merck-Serono, Novartis, Roche, Sanofi Genzyme, Takeda and Teva Pharmaceutical Industries, and receives research support from Biogen Idec, Merck-Serono, Novartis, Teva Pharmaceutical Industries, Roche, Italian Ministry of Health, Fondazione Italiana Sclerosi Multipla and ARiSLA (Fondazione Italiana di Ricerca per la SLA).

The remaining authors declare that the research was conducted in the absence of any commercial or financial relationships that could be construed as a potential conflict of interest.

## Publisher’s note

All claims expressed in this article are solely those of the authors and do not necessarily represent those of their affiliated organizations, or those of the publisher, the editors and the reviewers. Any product that may be evaluated in this article, or claim that may be made by its manufacturer, is not guaranteed or endorsed by the publisher.
