# Supplementary material for: Clinical and pathological findings in neurolymphomatosis: Preliminary association with gene expression profiles in sural nerves
Source: Front Oncol. 2022 Sep 26;12:974751. doi: 10.3389/fonc.2022.974751 (PMC9549065; doi:10.3389/fonc.2022.974751)
Supplement: Supplementary file 1 [file DataSheet_1.docx]

**Supplementary Information**

**Clinical and pathological findings in neurolymphomatosis: preliminary association with gene expression profiles in sural nerves.**

**Supplementary Figure 1**


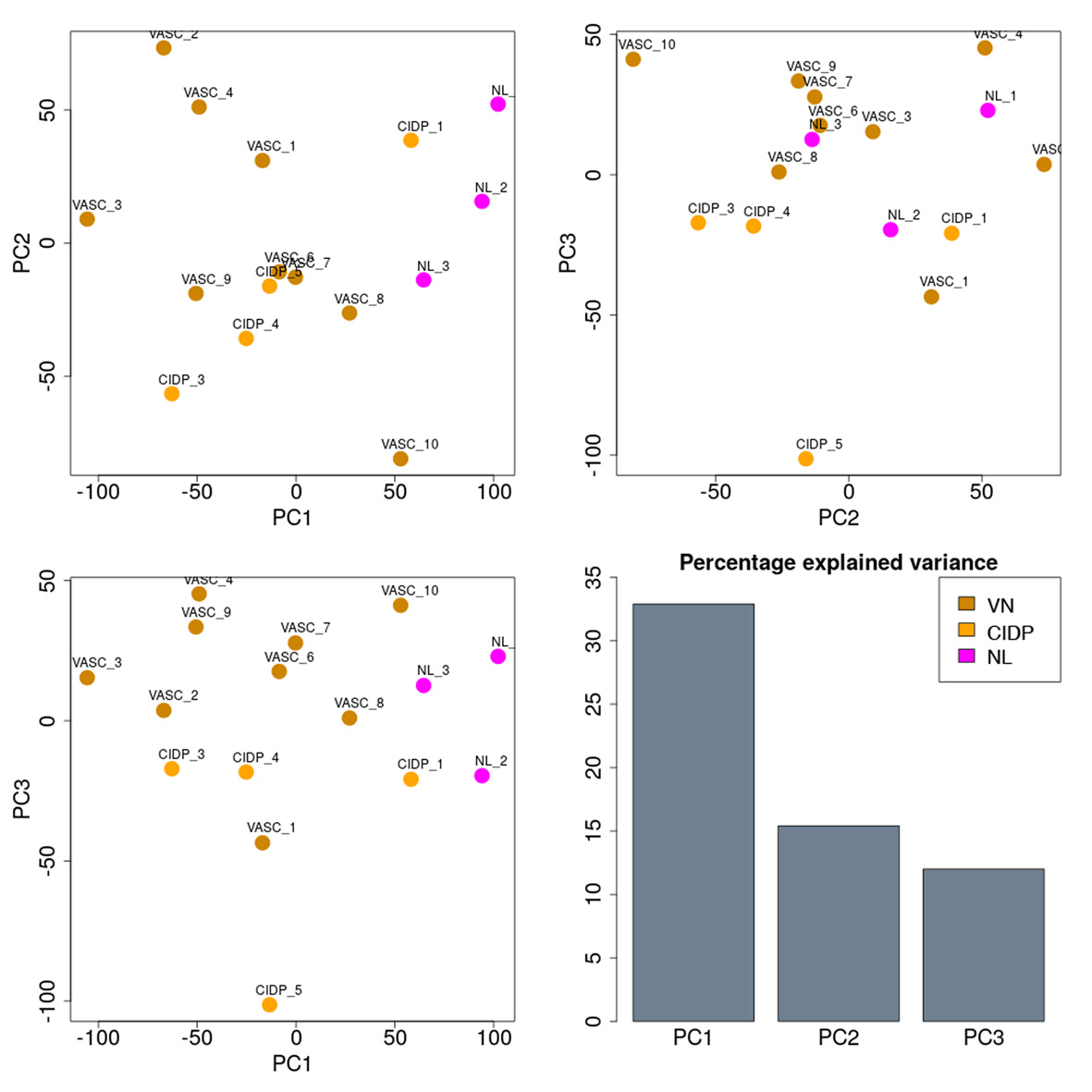


Principal Component (PC) score plot. Normalized expression values of the filtered probes for the 16 samples are projected onto the space defined by the combination of the first 3 PCs. A bar plot explained gene expression variance is reported in the bottom-left part of the figure.

**Supplementary Figure 2**


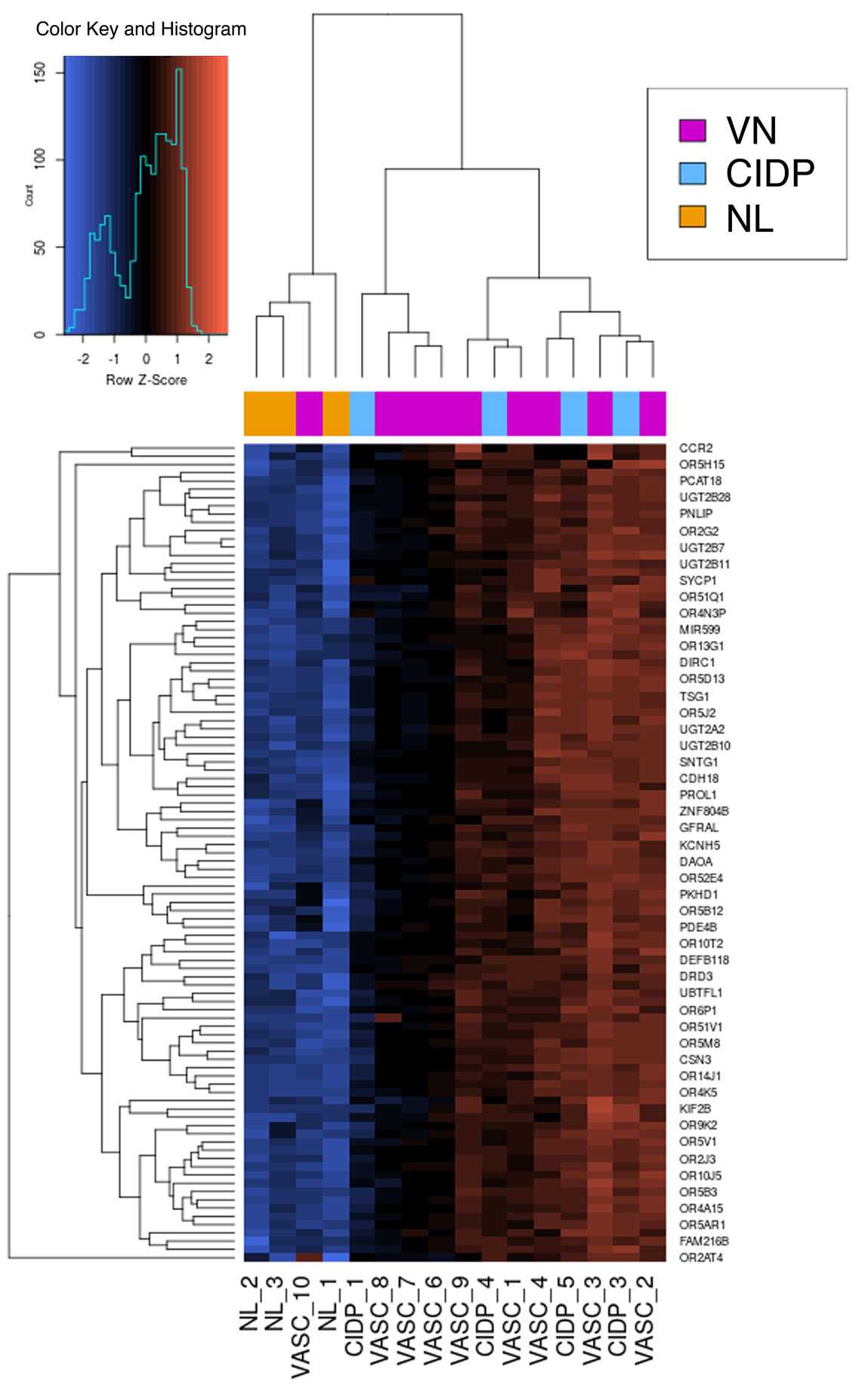


Heatmap of the down-regulated genes involved in neurolymphomatosis (NL).

**Supplementary Figure 3**

**
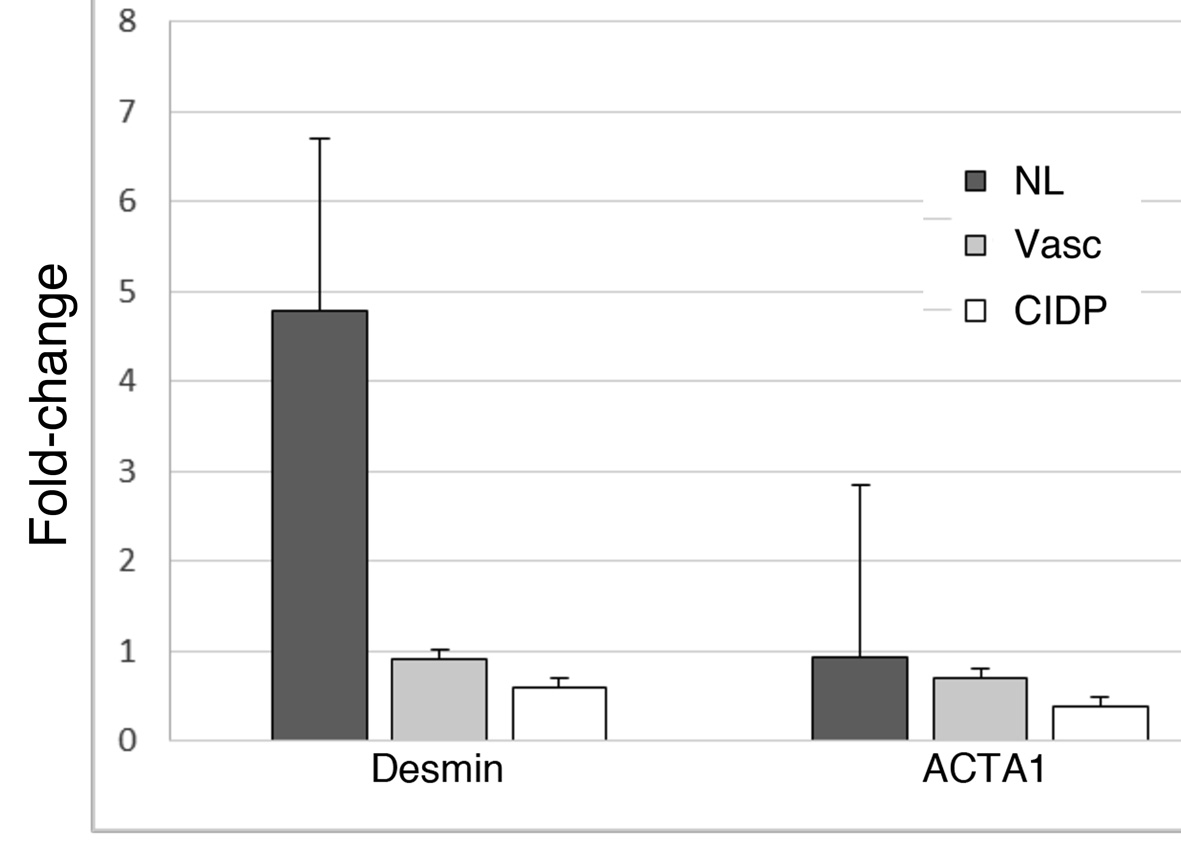
**

Validation by Real-time RT-PCR. Mean fold changes values (Bars: SEM) from the two selected up-regulated genes (*DES and ACTA1).*

**Supplementary Table 1. Pattern of expression for RPL26, RPS27 and RPS29**

| **Case** | NL1  (N°6) | NL2  (N°3) | NL3  (N°8) | NL4  (N°1) | NL5 (N°2) | NL6 (N°4) | CIDP1 (N°1) | CIDP2 (N°4) | VASC1 (N°1) | VASC2 (N°2) | VASC3 (N°8) | VASC4 (N°10) |
| --- | --- | --- | --- | --- | --- | --- | --- | --- | --- | --- | --- | --- |
|  | SMNP | MN | AMSAN | SMNP | MN | MN | CIDP | CIDP | VN | VN | VN | VN |
| **RPL26** |  |  |  |  |  |  |  |  |  |  |  |  |
| **Epineurium** |  |  |  |  |  |  |  |  |  |  |  |  |
| CD20 | +++ | + | 0 | ++ | 0 | 0 | 0 | 0 | 0/+ | 0 | 0/+ | 0 |
| CD3 | 0/+ | 0 | 0 | 0/+ | 0 | 0 | + | 0/+ | + | + | + | + |
| Vessels (EC) | + | + | + | + | + | + | + | + | + | + | + | + |
| **Endoneurium** |  |  |  |  |  |  |  |  |  |  |  |  |
| CD20 | 0/+ | +++ | +++ | 0 | ++ | +++ | 0 | 0 | 0 | 0 | 0 | 0 |
| CD3 | 0 | 0 | 0/+ | 0 | 0 | 0 | + | + | + | 0 | + | 0 |
| Vessels (EC) | + | ++ | +++ | + | ++ | ++ | + | + | + | + | + | + |
| Pericytes-like cells | + | ++ | +++ | 0 | + | + | 0/+ | 0 | 0 | 0 | 0 | 0 |
| Schwann cells | 0 | 0 | +++ | 0 | 0 | 0 | 0 | 0 | 0 | 0 | 0 | 0 |
| Axons | 0 | 0 | 0 | 0 | 0 | 0 | 0 | 0 | 0 | 0 | 0 | 0 |
| **RPS27** |  |  |  |  |  |  |  |  |  |  |  |  |
| **Epineurium** |  |  |  |  |  |  |  |  |  |  |  |  |
| CD20 | +++ | 0 | 0 | ++ | 0 | 0 | 0 | 0 | 0 | 0 | 0 | 0 |
| CD3 | 0/+ | 0 | 0 | 0 | 0 | 0 | 0 | 0 | + | + | 0/+ | 0/+ |
| Vessels (EC) | + | + | + | + | + | + | 0 | 0 | 0 | 0 | 0 | 0 |
| **Endoneurium** |  |  |  |  |  |  |  |  |  |  |  |  |
| CD20 | 0 | ++ | +++ | + | ++ | +++ | 0 | 0 | 0 | 0 | 0 | 0 |
| CD3 | 0 | 0 | 0/+ | 0 | 0 | 0 | 0 | 0 | 0 | 0 | 0 | 0 |
| Vessels | + | + | + | + | + | + | + | + | + | + | + | + |
| Pericytes-like cells | + | + | + | + | + | + | 0 | 0 | 0 | 0 | 0 | 0 |
| Schwann cells | + | + | + | + | + | 0 | 0 | 0 | 0 | 0 | 0 | 0 |
| Axons | ++ | ++ | + | + | ++ | + | 0 | 0 | 0 | 0 | 0 | 0 |
| **RPS29** |  |  |  |  |  |  |  |  |  |  |  |  |
| **Epineurium** |  |  |  |  |  |  |  |  |  |  |  |  |
| CD20 | +++ | 0 | 0 | ++ | 0 | 0 | 0 | 0 | 0 | 0 | 0 | 0 |
| CD3 | 0 | 0 | 0 | 0 | 0 | 0 | 0 | 0 | 0 | 0 | 0 | 0 |
| Vessels (EC) | + | + | + | + | + | + | 0 | 0 | 0 | 0 | 0 | 0 |
| **Endoneurium** |  |  |  |  |  |  |  |  |  |  |  |  |
| CD20 | 0 | ++ | +++ | 0 | ++ | +++ | 0 | 0 | 0 | 0 | 0 | 0 |
| CD3 | 0 | 0 | 0 | 0 | 0 | 0 | 0 | 0 | 0 | 0 | 0 | 0 |
| Vessels (EC) | 0/+ | 0/+ | 0/+ | 0/+ | 0/+ | 0/+ | 0 | 0 | 0 | 0 | 0 | 0 |
| Pericytes-like cells | 0 | 0 | 0 | 0 | 0 | 0 | 0 | 0 | 0 | 0 | 0 | 0 |
| Schwann cells | 0 | 0 | 0 | 0 | 0 | 0 | 0 | 0 | 0 | 0 | 0 | 0 |
| Axons | 0 | 0 | 0 | 0 | 0 | 0 | 0 | 0 | 0 | 0 | 0 | 0 |
| Neurolymphomatosis (NL), chronic inflammatory demyelinating polyneuropathy (CIDP), vasculitic neuropathy (VN). SMPN = sensory-motor polyneuropathy; MN = multiple mononeuropathy; AMSAN = acute motor-sensory axonal neuropathy; CD20 = B cell marker; CD3 = T cell marker; EC = endothelial cells. Immnoreactivity is graded from +++ (high number of positive cells) to 0 (absence of immunoreactivity). | | | | | | | | | | | | |
